# Characteristics of soil microbial community assembly patterns in fields with serious occurrence of tobacco Fusarium wilt disease

**DOI:** 10.3389/fmicb.2024.1482952

**Published:** 2024-11-13

**Authors:** Huidi Liu, Yongfeng Zhang, Hongchen Li, Shilu Chen, Jingze Zhang, Wei Ding

**Affiliations:** ^1^College of Plant Protection, Southwest University, Chongqing, China; ^2^Shangluo Prefecture Branch of Shaanxi Tobacco Corporation, Shangluo, China; ^3^Sanmenxia Tobacco Corporation of Henan Province, Sanmenxia, China; ^4^Institute of Biotechnology, Zhejiang University, Hangzhou, China

**Keywords:** Fusarium wilt disease, microbiome, microbiome assembly, microbial network, microbial interaction

## Abstract

**Introduction:**

Fusarium wilt disease (FWD) of tobacco is a destructive disease caused by *Fusarium* spp. in tobacco-growing regions worldwide. The *Fusarium* spp. infection may alter the composition and structure of the tobacco root microbial community; however, the relationship between these factors under large-scale geographical conditions in China remains underexplored.

**Methods:**

In the context of this investigation, soil samples from the rhizosphere of tobacco plants were procured from fields afflicted with FWD and those devoid of the disease in the Hanzhong region of Shaanxi province, as well as in the Sanmenxia and Nanyang regions of Henan province. These regions are recognized for the commercial cultivation of tobacco. The examination focused on discerning the influence of tobacco FWD on the composition and configuration of the rhizosphere microbial community, along with their co-occurrence patterns. This scrutiny was underpinned by targeted PCR amplification and high-throughput sequencing (amplicon sequencing) of the 16S rRNA gene and the ITS1 region.

**Results:**

The amplicon data analyses showed that FWD influenced the microbial structure and composition of the tobacco rhizosphere soil. FWD had a greater impact on the microbiome of the tobacco fungal community than on the microbiome of the bacterial community. Healthy plants had the ability to recruit potential beneficial bacteria. Diseased plants were more susceptible to colonization by other pathogenic fungi, but they still had the capacity to recruit potential beneficial bacteria. The analysis of microbial intra- and inter-kingdom networks further indicated that FWD destabilized microbial networks. In the overall microbial interaction, microorganisms primarily interacted within their boundaries, but FWD increased the proportion of interactions occurring across boundaries. In addition, FWD could disrupt the interactions within microbial network modules.

**Discussion:**

This study provides evidence that FWD can cause changes in the composition and network of microbial communities, affecting the interactions among various microorganisms, including bacteria and fungi. These findings contribute to our understanding of how plant microbiomes change due to disease. Furthermore, they add to our knowledge of the mechanisms that govern the assembly and interactions of microbial communities under the influence of FWD.

## 1 Introduction

Fusarium wilt of tobacco is a destructive plant disease, and it is caused by the soil-borne pathogen *Fusarium* spp. in tobacco-growing regions worldwide (Pandey, 2023). The *Fusarium* spp. lives as dormant chlamydospores in soil or mycelia on crop residues of infected plants (Toussoun et al., 1963). After overwintering, the fungi infect plant roots where the lateral root emerges from the primary root or in wounds. Once the vessels have been infected, the vascular tissues become blocked, resulting in the typical wilting (Boddy, 2016). More than seven species of *Fusarium*, which are known to infect tobacco plants, have been reported in China, namely *F. oxysporum* (Dean et al., 2012), *F. fujikuroi* (Shen et al., 2023), *F. tricinctum* (Qiu et al., 2023), *F. brachygibbosum* (Qiu et al., 2021), *F. meridionaleall* (Gai et al., 2023), *F. solani* (Yin et al., 2022), and *F. falciforme* (Qiu et al., 2022). The FWD occurrence caused serious economic losses in tobacco production in several provinces in China (Yao et al., 2021), and disease incidence ranges from 3 to 30% in Yunnan province (Gai et al., 2023).

An integrated approach involving resistance breeding, cultural measures, and biological and chemical controls is extensively used for effectively managing the FWD. However, the use of resistant varieties, the most efficient approach, may have a negative impact on the yield (Salameh et al., 2011; Li et al., 2020); cultural measures are limited, while the use of chemical fungicides carries the risk of pathogen resistance development (Lucas et al., 2015). By comparison, biological control is considered a promising alternative to fungicides, and understanding microorganism interactions in rhizosphere soil is fundamental to developing innovative biocontrol methods against plant pathogens (Mitter et al., 2019).

The rhizosphere, a complex and dynamic ecosystem, is home to a diverse microbial community (Chen et al., 2023). Microbial interactions in the rhizosphere environment can either suppress or promote plant diseases (Fitzpatrick et al., 2018). Microbial communities form intricate networks in the soil, building a biological barrier that facilitates microbe interactions and aids plant defenses against pathogen invasion near the root surface (Raaijmakers et al., 2009). Weibing Xun et al. used the “reductionist” and “strain knock-out” strategies to simplify the microbiota and demonstrate that keystone bacterial strains play an important role in maintaining plant health (Xun et al., 2023). While root microbiota are predominantly beneficial, certain members can act as pathogens, inducing plant diseases through mechanisms, thereby exerting detrimental effects on plant growth and overall performance (Fitzpatrick et al., 2018; Nadarajah and Abdul Rahman, 2021). The invasion of pathogens can affect strongly the relationships of the plant rhizosphere microbiome (Xiao et al., 2024). In the rhizosphere, soil microbes engage in interactions with pathogens, eliciting community responses that culminate in disease (Nobori et al., 2018). For instance, the non-pathogenic bacterial species *Erwinia toletana, Pantoea agglomerans*, and *Erwinia oleae* collaborate with the primary pathogen *Pseudomonas savastanoi* pv. savastanoi, exacerbating disease severity in olive trees (Buonaurio et al., 2015).

Studies have shown that Fusarium can alter the composition and function of plant rhizosphere microorganisms, thereby influencing the occurrence of plant diseases (Chang et al., 2021; Gao et al., 2021). *Fusarium* invasion influences the enrichment of specific beneficial microbial taxa in the plant rhizosphere, which may contribute to the plant's resistance to pathogen infection (Li et al., 2021). Symbiotic network analysis is increasingly utilized to deduce potential microbial interconnections and serves as a fundamental tool for gaining insights into microbial responses to pathogen invasions (Guimerà and Nunes Amaral, 2005). It is vitally important to our research into the potential interactions between microbiota and their responses to pathogen invasion. Currently, limited research has focused on the changes in tobacco rhizosphere microbial communities and their interactions after *Fusarium* invasion.

In this study, therefore, the rhizospheric soil samples of tobacco plants with FWD occurrence or without were collected from the tobacco-growing regions of Shaanxi and Henan provinces, China, where FWD incidence was high. We hypothesized that the disease occurrence influenced changes in the microbiome composition. To test this hypothesis, we explore the diversity and composition differences between the microbiomes of healthy and diseased plants based on the amplicon sequencing data. Moreover, the co-occurrence networks of healthy and diseased plant microbiomes were also compared, and this will provide new insight into the stability of communities as well as microbial succession under different environmental conditions.

## 2 Materials and methods

### 2.1 Sample collection

To compare the differences in soil microbial communities in rhizosphere soils associated with the occurrence of tobacco FWD vs. healthy plants, soil samples were collected in July 2023 during a serious occurrence of FWD (~75 days after tobacco transplanting). These samples were sourced from the primary tobacco tobacco-growing regions with similar latitudes north of the Yangtze River (Supplementary Table 1), where tobacco cultivation had persisted for over 20 years and FWD has been reported annually on the tobacco plants (cv. Yunyan99, a main cultivar). At each site, soil samples from tobacco plants that displayed no wilt symptoms were regarded as healthy ones, whereas from plants that showed wilt symptoms as diseased ones (O'Donnell et al., 1998). The rhizosphere soil (1 mm of soil tightly adhering to the root surface) at a depth of 10–20 cm for each sample was collected by vigorously shaking off the soil and brushing the remaining soil from the rhizosphere of a plant taproot. The samples wrapped in dry ice were taken to the laboratory and stored at −80°C until use (Edwards et al., 2015). Before soil sampling, disease incidence and severity were investigated. The disease assessment was carried out for each plant using a scale of 0–4, where 0 = healthy plants, 1 = stunted or off-color plants, 2 = plants with one symptomatic leaf, 3 = more than one symptomatic leaf, and 4 = dead plants (Lamondia, 1988; Thompson et al., 2011).

### 2.2 DNA extraction and amplicon sequencing

Total DNA was extracted from 0.5 g rhizosphere soil samples using the FastDNA Spin kit (MP Biomedicals, Santa Ana, CA, USA). PCR amplifications were conducted with primer pairs 515F (5′-GTGCCAGCMGCCGCGG-3′) and 806R (5′-GGACTACHVGGGTWTCTAAT-3′), which amplified the V3–V4 region of the 16S rRNA gene for bacteria (Shu et al., 2018). The primer pairs ITS1F (5′-CTTGGTCATTTAGAGGAAGTA-3′) and ITS2R (5′-GCTGCGTTCTTCATCGATGC-3′) were applied to amplify the ITS region for fungi (Adams et al., 2013). The amplification was performed in a 20-μL reaction volume, which contained 10 μL of 2 × Pro Taq, 0.8 μL of each primer (5 μM), 0.4 μL of fast Pfu polymerase, 10 ng of template DNA, and ddH_2_O. The thermal cycling program was performed with 30 cycles after an initial denaturation at 95°C for 4 min. Each cycle included a denaturation step at 95°C for 30 s, annealing at 50 or 55°C for 1 min for 16S gene or ITS region sequences, and an extension step at 72°C for 45 s. PCR products were purified from a 2% agarose gel using a PCR Clean-Up Kit (YuHua, Shanghai, China) and quantified with a Qubit 4.0 fluorometer (Thermo Fisher Scientific, Waltham, MA, USA). The soil microbial diversity was sequenced using the Illumina Miseq PE300 platform (Illumina, San Diego, CA, USA). The purified amplicons were initially assembled according to the standard of equimolar amounts and then subjected to paired-end sequencing.

The raw data were de-multiplexed and then quality filtered using fastp version 0.19.6 (Chen et al., 2018). The overlapping paired-end reads were merged to a reference standard using FLASH version 1.2.11 (Magoč and Salzberg, 2011). Next, the sequence and abundance information represented by the amplicon sequence variant (ASV) was obtained by processing the data using the sequence denoising method (DADA2) plugin in the QIIME2 (version 2020.2; Bolyen et al., 2019).

The raw sequencing reads were deposited into the NCBI Sequence Read Archive (SRA) database (Accession Number: PRJNA1148747).

### 2.3 Bioinformatics analysis

The microbial α- and β-diversity analyses were carried out using the Majorbio Cloud (http://www.majorbio.com), which was a one-stop online analytic platform, and the dilution curve and α- and β-diversity indices were calculated. The Bray–Curtis distance matrix served as the basis for principal coordinate analysis (PCoA) for evaluating the dissimilarity of the microbial communities. With 999 permutations and the Bray–Curtis distance matrix as an input, permutational multivariate analysis of variance (PERMANOVA) statistical tests were run in the *vegan* package with the function “adonis” to ascertain the effects of different factors on the community dissimilarity (Nakagawa and Schielzeth, 2013). Species differences were assessed using linear discriminant analysis effect size (LEfSe) across multiple taxonomic levels (phylum, class, order, family, and genus) in the rhizosphere soil of healthy and diseased plants. LDA values were used to measure the impact of different species, showing significant differences between healthy and diseased soils in relation to the FWD. This analysis indicates that different taxa play a key role in the process of tobacco FWD (Chang et al., 2022).

### 2.4 Microbial network construction

The microbial networks' statistical analyses were conducted in the R environment (v4.3.2; http://www.r-project.org/). Microbial association networks were created by removing fungal and bacterial OTUs with relative abundances <0.01% from the dataset (Faust, 2021). The OTUs appeared to be less than one-fifth in all samples and were removed to avoid random effects of rare and site-specific microbiomes. Robust correlations based on Spearman's correlation coefficients (ρ) of >0.6 or <-0.6 and false discovery rate-corrected *p*-values <0.001 were used to construct networks (Jiao et al., 2022). The networks were visualized using the interactive platform Gephi software (version 0.10; Bastian et al., 2009). Network visualization was visualized using Cytoscape software (version 3.9.1; Zhang et al., 2021).

From these networks, the network's fundamental information, including the number of nodes, edges, positive correlations, and negative correlations, was obtained. The number of links was counted, and complexity was calculated as linkage density (links per OTU) among bacterial, fungal, fungi–bacterial only, or all fungal and bacterial OTUs (Gao et al., 2021). The OTUs with the top 10 nodes with the highest degree and closeness centrality were identified, and network stability was evaluated by the proportion of negative or positive correlations and the modularity (Newman, 2006; Olesen et al., 2007).

## 3 Results

### 3.1 FWD affects the tobacco rhizosphere microbiome assembly

**Figure 1:**
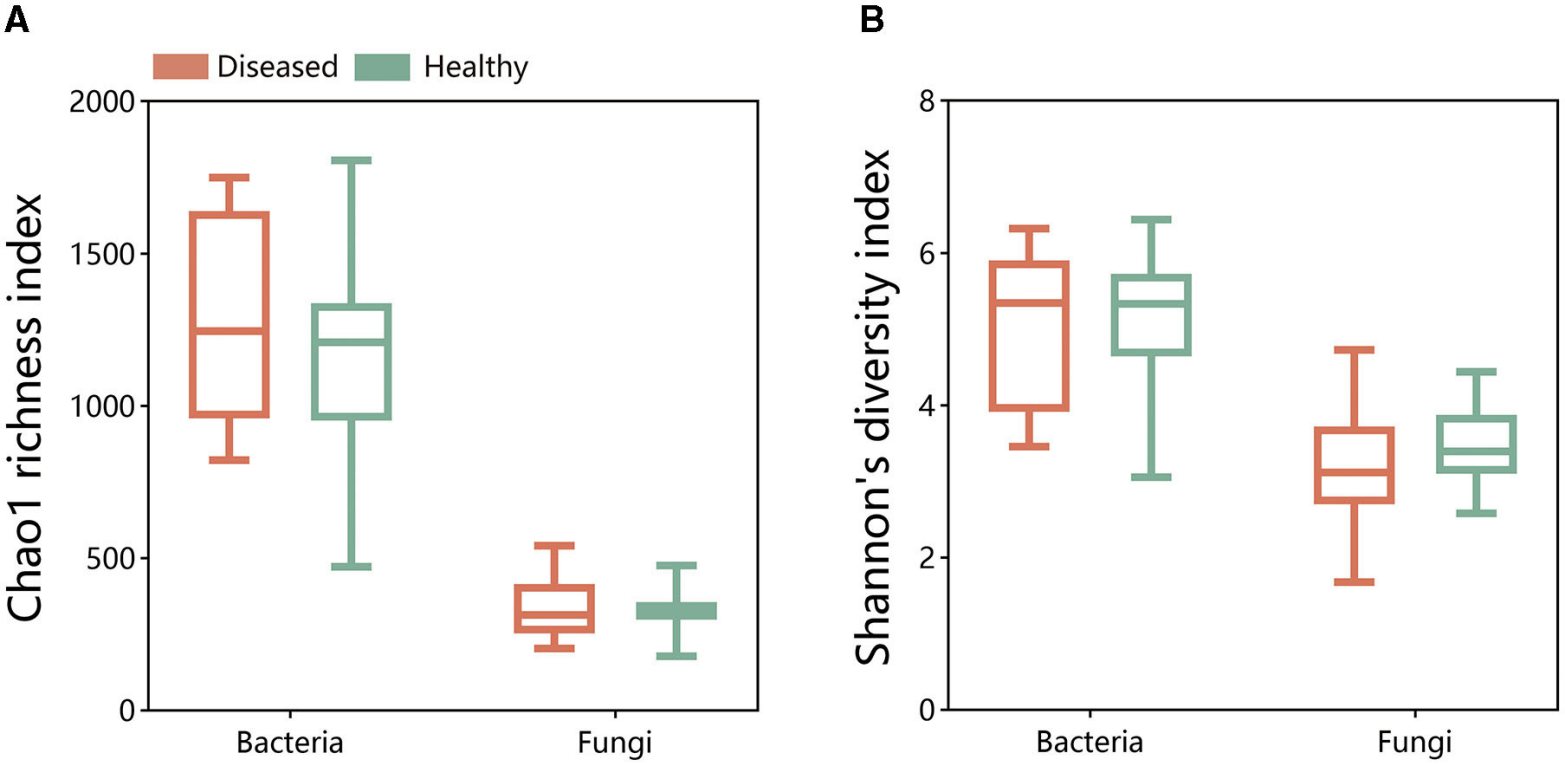
Chao1 richness index and Shannon's diversity index of bacterial **(A)** and fungal **(B)** communities in the disease and health plants of rhizosphere soil.

A comparison of the alpha diversity of bacterial and fungal microbial communities using the Chao1 richness index and Shannon's diversity index revealed no significant differences between the alpha diversity of diseased and healthy tobacco rhizosphere microbiomes (Figure 1). Meanwhile, there was no significant difference in samples collected from Sanmenxia and Neixiang regions (Supplementary Figure 1). The results indicated that FWD had no statistically significant difference in the microbial community in tobacco plants.

**Figure 2:**
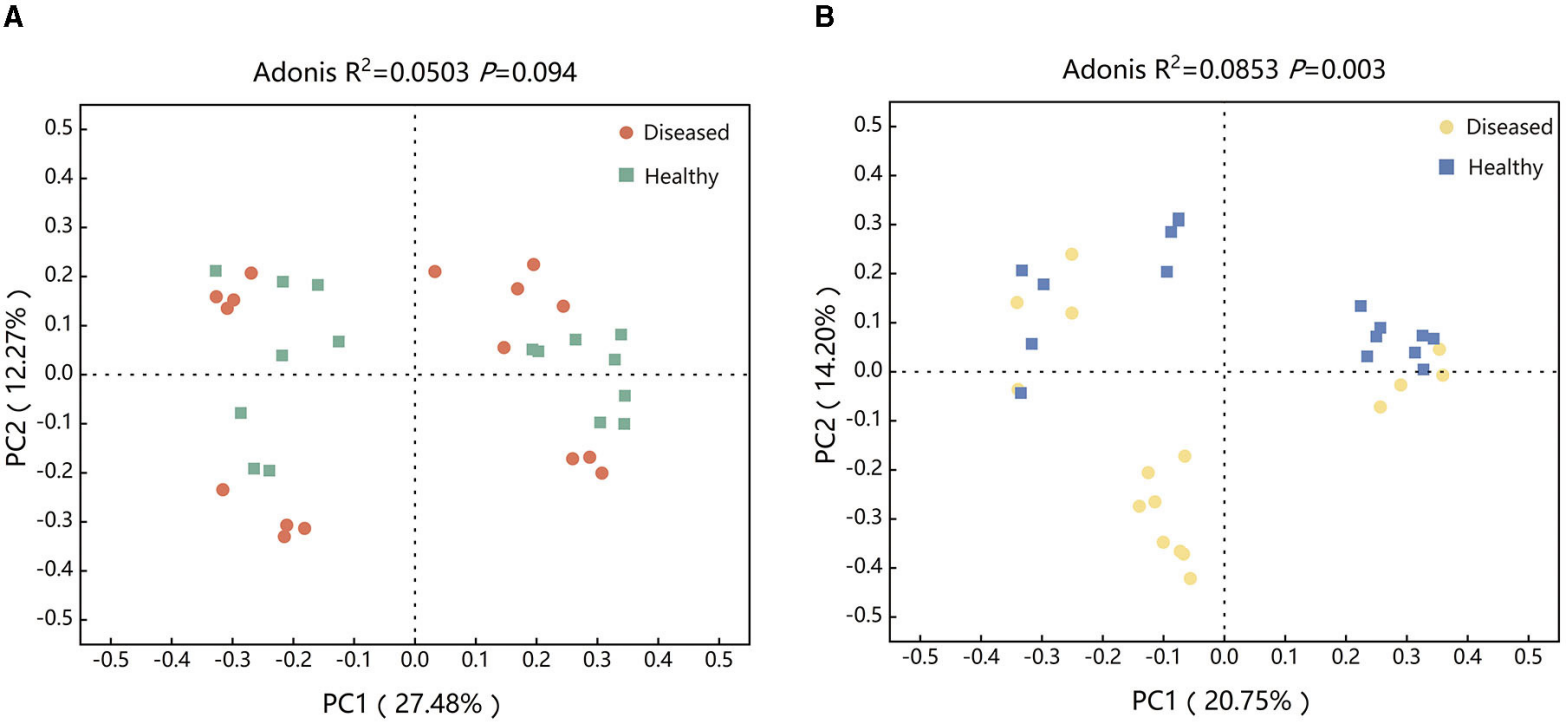
Structure of tobacco bacterial **(A)** and fungal **(B)** communities at different periods. **(A)** Principal coordinates analysis (PCoA) ordinations of Bray–Cutis dissimilarity matrices show a significant association of the bacterial microbial community composition affected by FWD. **(B)** PCoA ordinations of Bray–Cutis dissimilarity matrices show a significant association of the fungal microbial community composition affected by FWD.

To examine the effect of FWD on tobacco rhizosphere soil microbiome, PCoA and Adonis analysis were used for evaluating the diseased and healthy microbial community structure. The results found that there was no statistically significant difference in the bacterial microbial community structures between healthy and diseased tobacco infected by FWD (*R*^2^ = 0.0503, *P* = 0.094; Figure 2A). However, there were significant differences in the fungi microbial community structures (*R*^2^ = 0.0853, *P* = 0.003; Figure 2B, Supplementary Figure 2). The bacterial (*R*^2^ = 0.4722, *P* = 0.027) and fungal community (*R*^2^ = 0.4458, *P* = 0.027) structures of the Huamiao Village and Shangluo block exhibited significant differences under the influence of FWD. The bacterial (*R*^2^ = 0.5398, *P* = 0.027) and fungal community (*R*^2^ = 0.5832, *P* = 0.027) structures of the Dragon King Ridge, Shangluo block exhibited significant differences under the influence of FWD. The bacterial (*R*^2^ = 0.3583, *P* = 0.027) and fungal community (*R*^2^ = 0.3798, *P* = 0.027) structures of the Sanmenxia block exhibited significant differences under the influence of FWD. The bacterial (*R*^2^ = 0.5034, *P* = 0.027) and fungal community (*R*^2^ = 0.5741, *P* = 0.027) structures of the Neixiang block exhibited significant differences under the influence of FWD. The bacterial and fungal populations at various sampling sites differed significantly under the influence of FWD.

**Figure 3:**
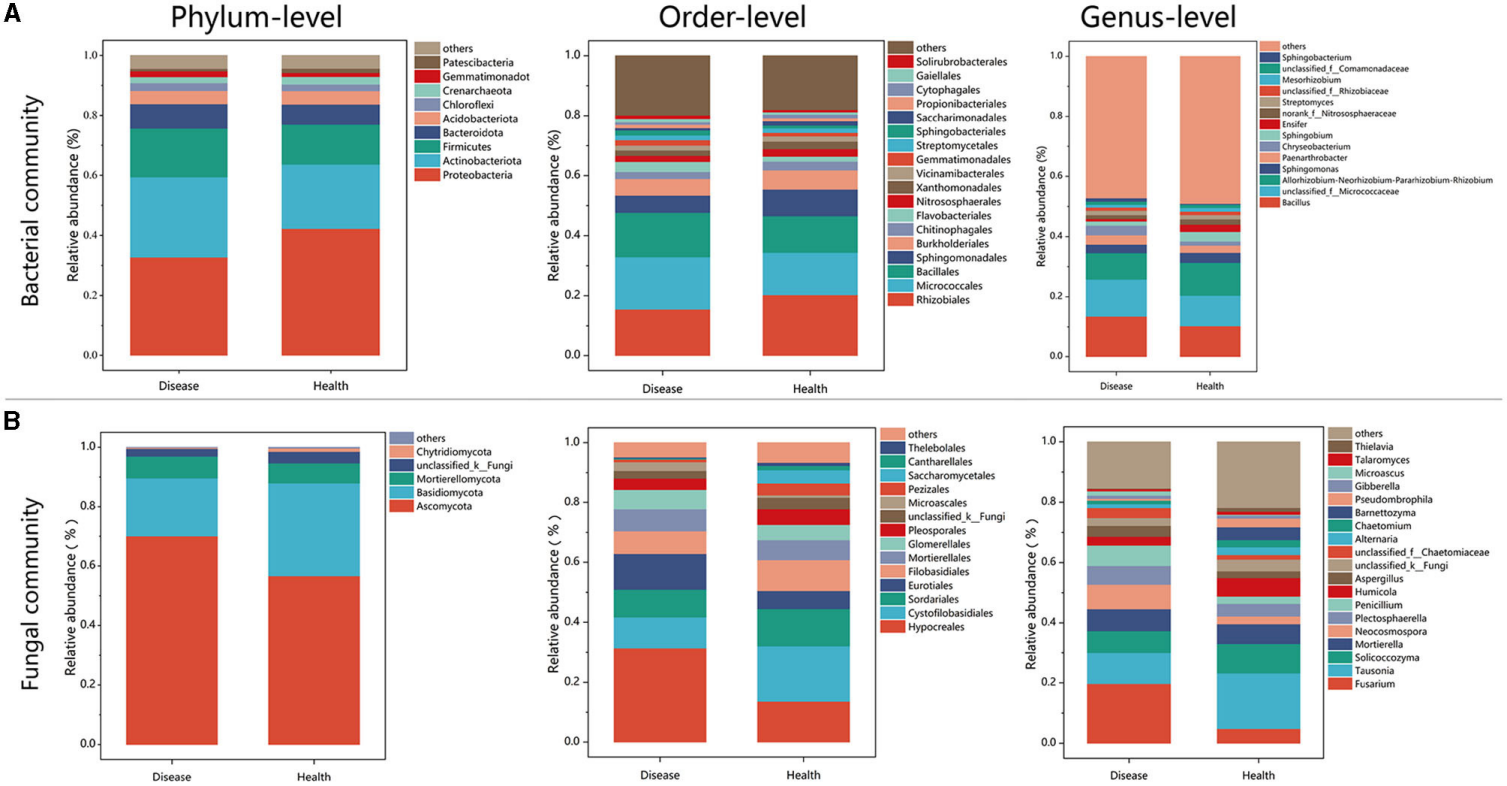
Bacterial and fungal community structures in diseased and healthy soils. **(A)** Bacterial rhizosphere soil community structures. **(B)** Fungal rhizosphere soil community structures.

**Figure 4:**
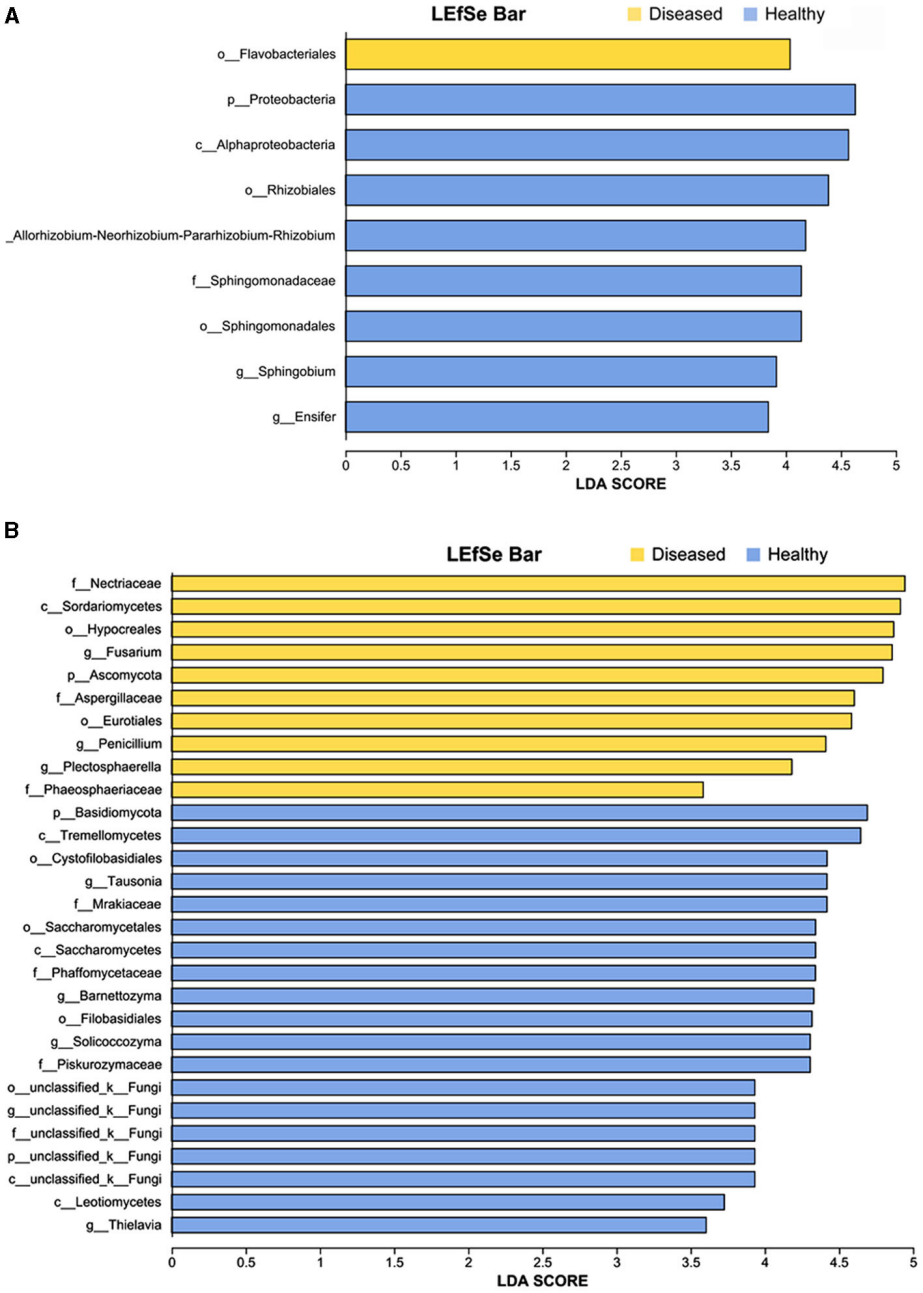
LEfSe analysis of bacteria **(A)** and fungi **(B)** whose abundances significantly differed between the diseased and healthy samples. The findings with regards to microbiome from phylum to genus are shown (LDA score > 3.5, *p* < 0.05).

There were notable variations in the bacterial community composition between the healthy and diseased soil samples, according to the data on the phylum-level composition of these two types of soil (Figure 3). *Proteobacteria* was the most dominant bacterial phylum in soil samples, followed by *Actinobacteria*. The relative abundance of *Proteobacteria* in healthy plants was 42.26%, significantly higher than the 32.74% observed in diseased plants (*P* < 0.001; Figure 3A, Supplementary Figure 3A). Relative abundance of the top five bacteria from healthy (H) and diseased (D) samples on the order level were *Rhizobiales* (H:20.30%; D:15.52%), *Micrococcales* (H:14.10%; D:17.41%), *Bacillales* (H:12.16%; D:14.75%), *Sphingomonadales* (H:8.87%; D:5.73%), and *Burkholderiales* (H:6.38%; D:5.52%). Among them, *Rhizobiales, Sphingomonadales*, and *Burkholderiales* belonged to *Proteobacteria, Micrococcales* belonged to *Actinobacteriota*, and *Bacillales* belonged to Firmicutes (Figure 3A). In fungal communities enriched in the soil, the most dominant taxa were *Ascomycota* and *Basidiomycota*. The relative abundance of taxa in Ascomycota in the diseased samples is higher than that in healthy plants. However, the relative abundance of taxa in *Basidiomycota* in the healthy samples (31.30%) was significantly higher than that in diseased samples (19.58%; *P* < 0.05; Figure 3B, Supplementary Figure 3B). Relative abundance of the top five fungal orders for samples collected from healthy and diseased tobacco plants were *Hypocreales* (H:13.65%; D:31.39%), *Cytofilobasidiales* (H:18.44%; D:10.33%), *Sordariales* (H:12.39%; D:9.25%), *Eurotiales* (H:6.03%; D:11.90%), and *Filobasidiales* (H:10.26%; D:7.59%). The results display genera in fungal communities with relative abundances larger than 0.01% (Figure 4B). The relative abundance of *Fusarium* reached 19.73% in diseased tobacco rhizosphere, which was significantly higher than healthy tobacco plants with 4.83% (*P* < 0.01; Figure 3B, Supplementary Figure 3C).

### 3.2 Rhizosphere microbial taxa discriminated by soil conditions

The linear discriminant analysis (LDA) effect size (LEfSe) method was used to detect bacterial and fungal taxa causing significant differences under the influence of FWD. The results showed that several genera of beneficial bacteria belonging to *Proteobacteria* (LDA = 4.04), such as *Rhizobium* (LDA = 4.118), *Sphingobium* (LDA = 3.91), and *Ensifer* (LDA = 3.84) were enriched in the healthy tobacco plants, namely their abundances were significantly higher in the healthy tobacco plants than that in the diseased plants (Figure 4A, Supplementary Table 2). Similarly, several genera of fungi belonging to *Ascomycota* (LDA = 4.80) such as *Fusarium* (LDA = 4.86), *Penicillium* (LDA = 4.41), *Plectosphaerella* (LDA = 4.18), and *Thielavia* (LDA = 3.60), were enriched in the diseased tobacco plants. Among them, Fusarium species possibly included pathogen species because the fungal community was more affected by *Fusarium* than the bacterial community (Supplementary Table 3).

### 3.3 Effect of FWD tobacco microbiome intra-kingdom networks

**Figure 5:**
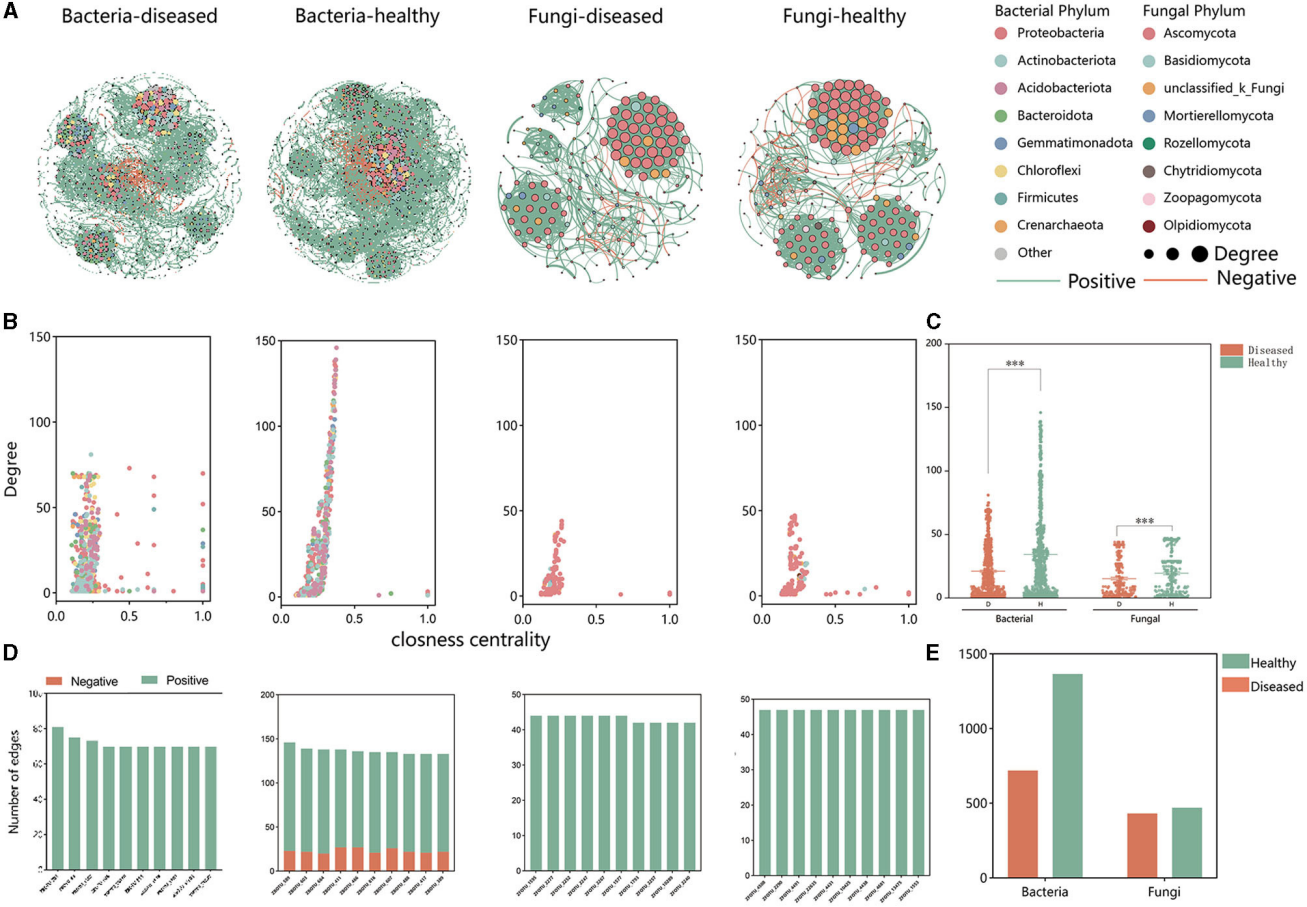
Intra-kingdom co-occurrence networks. **(A)** Bacterial and fungal co-occurrence networks in healthy and FWD plants. Node represents different bacterial and fungal phylum by different colors. Node size indicates the degree of connection. Edges represent the pairwise correlations between the nodes in a network, and their colors represent positive (green) and negative (red) correlations. **(B)** A comparison of node-level topological features (degree and closeness centrality) displays the high degree and closeness centrality for the hub taxa. **(C)** The degree of bacterial and fungal taxa shows the higher complexity of the healthy bacterial network than that of the diseased bacterial network. The significance of the difference was determined by Fisher's LSD test (***P < 0.001). **(D)** Degree and interaction type of the top 10 hub nodes in four networks. **(E)** Significance of difference in edges.

Based on intra-kingdom co-occurrence network analysis, a higher proportion of negative edges and modularity in the bacterial network (proportion of negative edges and modularity: 14.26%/0.503 in healthy and 2.34%/0.737 in FWD) than in the fungal networks (proportion of negative edges and modularity: 3.28%/0.641 in healthy conditions and 1.56%/0.574 in FWD) was revealed. A higher number of nodes and edges in the bacterial networks than in the fungal networks were recorded, while the health networks had more nodes and edges than the disease networks in bacterial and fungal networks (Supplementary Table 4). In addition, the bacterial healthy microbiome networks exhibited greater negative connections than the fungal healthy networks (Figure 5A) and demonstrated higher degrees of closeness within the microbiota community (Figures 5B, C). Moreover, in the bacterial networks, the healthy networks have many more edges than the disease networks. The edges of the top 10 hub nodes with high degree and proximity centrality values were predominantly negative with other nodes in the healthy networks. By comparison, the fungal networks did not have a negative correlation in the top 10 hub nodes (Figures 5D, E, Supplementary Table 5).

### 3.4 Effect of FWD on tobacco microbiome inter-kingdom co-occurrence networks

**Figure 6:**
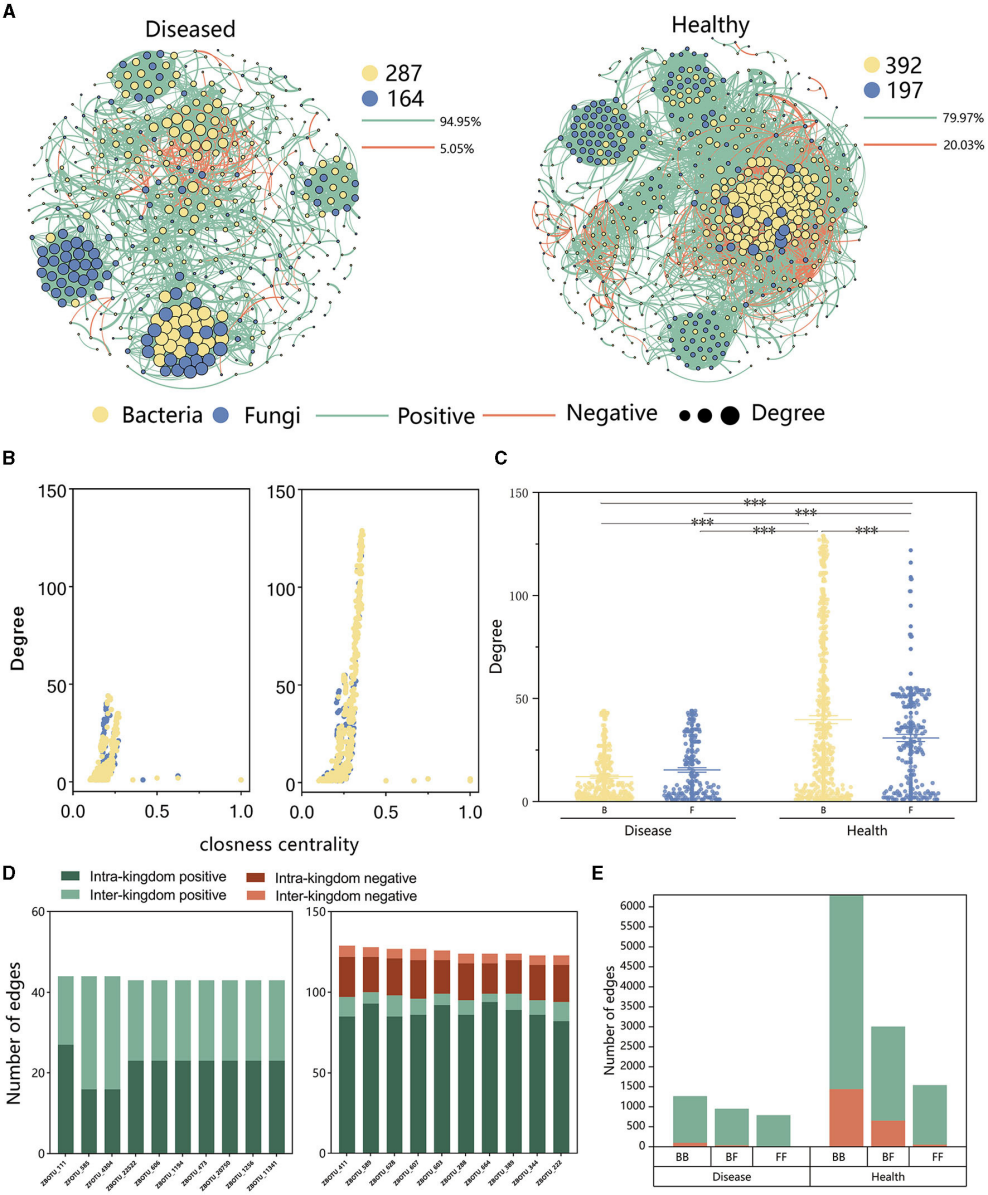
Inter-kingdom co-occurrence networks. **(A)** Network contained both bacterial and fungal taxa and showed a higher number of fungal taxa and a lower number of bacterial taxa in the diseased network than those in the healthy network. **(B)** A comparison of node-level topological features in **(A)** (degree and closeness centrality) demonstrates the high degree and closeness centrality values for the hub taxa. **(C)** Degree values of bacterial and fungal taxa in healthy and diseased networks. The significance of the difference was determined using Fisher's LSD test (***P < 0.001). **(D)** Degree and interaction type of the top 10 hub nodes in diseased (left) and healthy (right) networks. “Intra-kingdom correlation” refers to BB or FF, and “interkingdom correlation” refers to BF. **(E)** Number of bacterial–bacterial (BB), bacterial–fungal (BF), and fungal–fungal (FF) correlations in the healthy and diseased networks. The green and red colors of the edges and columns indicate positive and negative correlations, respectively.

Analysis of the inter-kingdom co-occurrence network and co-occurrence patterns of bacteria and fungi showed that variations in these networks and patterns in soil microbiomes were associated with the occurrence of FWD. The proportion of negative edges and modularity was higher in healthy networks (proportion of negative edges/modularity: 20.03%/0.534) than in the diseased networks (proportion of negative edges/modularity: 5.05%/0.752). There were more bacterial nodes in the inter-kingdom co-occurrence networks in both diseased and healthy plants (Figure 6A), and positive correlation was higher in diseased plants than in healthy plants, indicating that FWD possibly destabilized the network. In addition, the healthy network had a higher degree and closeness nodes, and among them, the number of bacterial nodes was higher than fungal ones (Figures 6B, C). The top 10 hub taxa were bacteria in the healthy network, while fungal taxa accounted for 2 hubs in the diseased plant network (Figure 6D, Supplementary Table 5). ZFOTU_585 was among the top 10 hub nodes in inter-kingdom co-occurrence disease networks, which belonged to the genus *Fusarium*. Inter-kingdom and intra-kingdom negative correlations were higher in the diseased plants than in the healthy plants (Figure 6D, Supplementary Figure 4). Upon analyzing the edges of the networks, we found that the proportion of bacterial–fungal (BF) inter-kingdom edges in the disease network (31.59%) was higher than that in the healthy network (27.73%). The healthy network exhibited a higher proportion of negative correlations (28.19%), whereas the disease network showed a lower proportion (4.66%), displaying that FWD enhanced bacteria–fungus interactions (Figure 6E). The results also indicated that the bacterial–bacterial (BB), bacterial–fungal (BF) inter-kingdom, and fungal–fungal (FF) networks had more negative correlations in the healthy network.

### 3.5 Characteristics of network modularity in inter-kingdom co-occurrence networks

**Figure 7:**
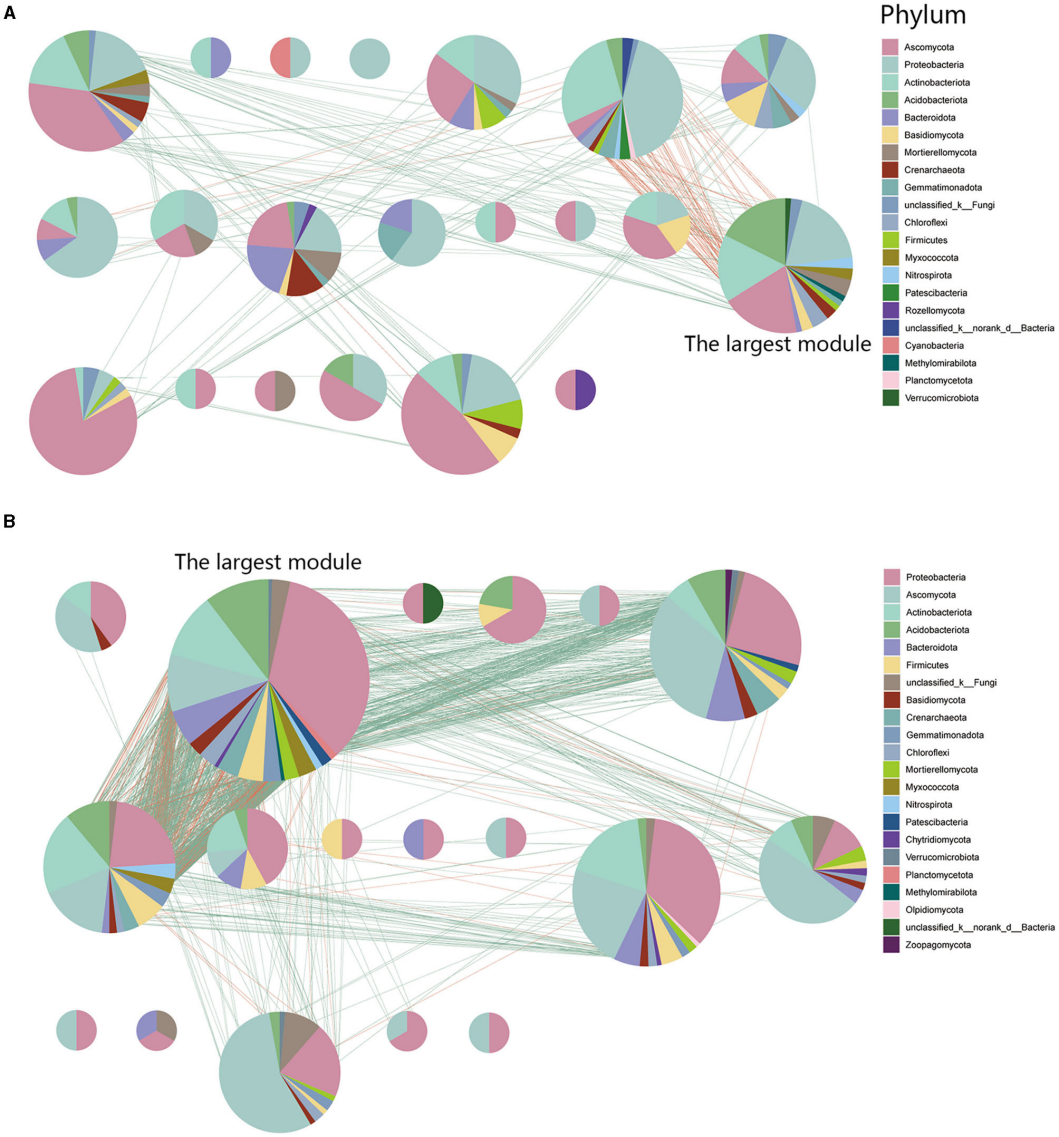
Network modularity in inter-kingdom co-occurrence networks. **(A)** Modules in diseased inter-kingdom co-occurrence networks. **(B)** Modules in healthy inter-kingdom co-occurrence networks. A red link indicates a negative correlation between two individual nodes, whereas a green link indicates a positive correlation.

To identify potential interactions within the rhizosphere soil microbiome, we performed modularization of the inter-kingdom co-occurrence networks. Analytical results indicated that FWD could alter microbial interactions and composition within microbial network modules. Nodes within the diseased microbial network (93.61%) exhibit more interactions in internal modules, whereas in the healthy network (85.69%), the trend is reversed. The results displayed that there were more modules in the diseased network and the largest module had fewer nodes and edges (nodes:16.41%; edges:12.82%) than healthy network (nodes:29.37%; edges:60.28%; Figures 7A, B). At the phylum level, the largest modules in the inter-kingdom co-occurrence networks between diseased and healthy communities had different compositions. The healthy networks have more *Proteobacterial* microbes, and their microbial associations are more complex (Figure 7B).

## 4 Discussion

### 4.1 Effect of FWD on rhizosphere microbial composition of tobacco

The rhizosphere microbiome plays an important role in plant health. Understanding the differences in microbial communities between diseased and healthy plants in rhizosphere soils as well as investigating key taxa and their correlations are necessary for exploring the interactions between plants and pathogens (Huang et al., 2022). In this study, significant differences were found in the microbiomes between the diseased (FWD) and healthy rhizosphere soils (Figure 2). Usually, plants provide nutrients and niches for microbes; in return, these microbes play pivotal roles in promoting plant growth and tolerance against biotic and abiotic stresses and forming a barrier against the invasion of pathogens. For instance, species of *Chitinophaga, Flavobacterium*, and *Pseudomonas* were enriched with sugar beet to suppress the root pathogen *Rhizoctonia solani* (Carrión et al., 2019). Recent studies have proven that *flavobacterium* is significantly associated with Fe (III)_Hcl_, potentially influencing multiple nitrogen cycle variables, and proving useful for the root sheath of barley grown in acidic soil (Yu et al., 2021; Xu et al., 2023). The abundance of *Rhizobium* and *Sphingomonas* was positively correlated with nitrogen fixation (Liu et al., 2023). This can enhance the plant's nitrogen utilization and consequently promote plant health (Vries et al., 2022). Our results also showed diversity and abundance of beneficial microbes were higher in healthy tobacco plants than in diseased plants (Figure 4). Beneficial flora indirectly enhances plant growth and nutrient uptake by modifying the structure and function of native rhizosphere microbial communities (Hu et al., 2021; Pang et al., 2021).

However, pathogen infestation may cause changes in the composition of plant root secretions and in the structure of biological communities. In this study, The LEfSe analysis revealed that genera of beneficial bacteria *Flavobacterium, Rhizobium, Sphingobium*, and *Ensifer* were enriched in the diseased and healthy tobacco plant rhizosphere, but their abundances were significantly higher in the healthy tobacco plants than that in the diseased plants. Colonization and host infestation of pathogenic *Fusarium* spp. causing FWD in soil can influence changes in the structure of rhizosphere microbial communities. In addition, few studies showed that plants with FWD have more lateral roots (Silva Lima et al., 2019). The *flavobacterium* enriched in diseased rhizosphere soil may be associated with the generation of more lateral roots in tobacco plants. Therefore, these results displayed that changes in diversity and abundance of beneficial microbes in the bacterial and fungal community structures were associated with diseased and healthy tobacco plants. Analyses of diseased and healthy microbiome structures provide evidence for soil-beneficial microorganisms that maintain plant health; it is necessary to conduct culture-based experiments to prove the hypothesis. Specifically, it is important to isolate potentially beneficial bacteria that are enriched in the rhizosphere soil and evaluate their antifungal activity against pathogens.

### 4.2 Effect of FWD on the rhizosphere microbial network structure of tobacco

According to theoretical modeling and simulations, microbial networks with lower positive correlations and higher negative correlations among members are more stable (Coyte et al., 2015; Hernandez et al., 2021). The healthy microbial network demonstrated a higher percentage of negative correlations than the diseased microbial network, including its core taxa, except for the fungi intra-kingdom co-occurrence network. In this study, we found that pathogenic fungi in the health network have stronger negative correlations with other microorganisms. The stability of healthy microbial networks may be maintained by bacteria that compete with pathogenic fungi for access to the same root secretions (Huang et al., 2017). The proportion of interactions between bacteria and fungi in the pathogenic network increased. *Burkholderia glumae*, a seed-borne plant pathogenic bacterium, and *Fusarium graminearum*, an air-borne plant pathogenic fungus, interact to enhance bacterial survival, dispersal of both bacteria and fungi, and promote disease progression on rice plants (Ruiz-Bedoya et al., 2023). The interaction between bacteria and fungi in the disease network can potentially facilitate the dissemination of *Fusarium* and contribute to the progression of FWD. In the interaction network, bacteria occupy more nodes and correlations. The top 10 nodes in the health network consist solely of bacteria, whereas the disease network has fungi. Bacteria were more dominant in the healthy network than in the diseased network. The findings indicate that bacteria are the predominant group in the soil, indicating their potential role in preserving soil health.

### 4.3 Effect of FWD on the modularity of microbial networks

Community stability is influenced by modularity and microbial interactions (Coyte et al., 2015). In this study, the modularity of the disease network exceeded that of the health network. The analyzed results also indicated that the connections among modules in the health network are more intricate, whereas the connections within modules in the disease network are more complex. In microbial networks, there is a high probability that different modules have different functions in the ecological niche. A healthy microbiome may promote interactions between different functional microbes to maintain plant health (Fan et al., 2019). Furthermore, we conducted network analysis to visualize modules with high interactions. Notably, the largest module observed in the network was deemed as the central module in our study (Huang et al., 2019). This central module serves as a crucial hub of microbial interactions, likely playing a significant role in shaping the overall microbial community dynamics and subsequently influencing plant health. By focusing on this central module, our analysis revealed that the central module in the health network exhibited a higher number of nodes and edges than the other modules. In addition, Proteobacteria occupy a higher proportion in the largest module of the health network than the pathogenic network. Several potentially beneficial microorganisms belong to the Proteobacteria. Microdiversity can result in the emergence of distinct ecotypes within a single species. These ecotypes are hypothesized to confer temporal and spatial stability to the species. In previous research, we found that ecotypes within the Proteobacteria shared the same OTUs but exhibited different distribution patterns across space and time (García-García et al., 2019). These microorganisms may play a crucial role in strengthening the interrelationship between the network structures and key microbial community functions, thereby contributing to the maintenance of network stability (Yuan et al., 2021).

## 5 Conclusion

In the present study, we reported the effect of FWD occurrence on the composition and structure of the tobacco root microbial community in diseased tobacco plants compared to healthy plants. The amplicon data analysis showed that FWD influenced the microbial structure and composition of the tobacco rhizosphere soil. Several genera of potentially pathogenic fungi, including *Fusarium, Penicillium, Plectosphaerella*, and *Thielavia* from the *Ascomycota* phylum, were enriched in diseased tobacco rhizosphere soils. Potentially beneficial bacteria, such as *Rhizobium, Sphingobium*, and *Ensifer* from the Proteobacteria phylum, were enriched in healthy tobacco rhizosphere soils. Bacteria occupy a crucial position in microbial networks, and their interactions with pathogens play a vital role in sustaining healthy microbial ecosystems. The current study provides a good understanding of microbiome assembly and function under FWD and reveals the potential for utilizing soil microbiomes to promote plant health and sustainable agricultural production.

## Data Availability

The datasets presented in this study can be found in online repositories. The names of the repository/repositories and accession number(s) can be found at: https://www.ncbi.nlm.nih.gov/, PRJNA1148747.
