# Supplementary material for: Characteristics of soil microbial community assembly patterns in fields with serious occurrence of tobacco Fusarium wilt disease
Source: Front Microbiol. 2024 Nov 13;15:1482952. doi: 10.3389/fmicb.2024.1482952 (PMC11600729; doi:10.3389/fmicb.2024.1482952)
Supplement: Supplementary file 1 [file Data_Sheet_1.pdf]

Supplementary Material

Characteristics of Soil Microbial Community Assembly Patterns in fields with Serious occurrence of Tobacco *Fusarium* Wilt Disease

Huidi Liu[1], Yongfeng Zhang[2], Hongchen Li[3], Shilu Chen[1], Jingze Zhang[4*] & Wei Ding[1*]

[1] College of Plant Protection, Southwest university, Chongqing 400715, China;

[2] Shangluo Prefecture Branch of Shaanxi Tobacco Corporation, Shangluo 726099, China;

[3] Sanmenxia Tobacco Corporation of Henan province, Sanmenxia 472000, China;

[4] College of Plant Protection, Zhejiang University, Hangzhou 310000, China.

List of supporting information:

Fig.S1 Chao1 richness index and Shannon's diversity index of bacterial and fungal communities affected by FWD in the different sampling site.

Fig.S2 Principal coordinates analysis (PCoA) ordinations of Bray–Cutis dissimilarity matrices showing a significant association of the bacterial (up) and fungi (under) disease and health community composition in different sites.

Fig.S3 The percents of Prteobacteria (a), Basidiomycota (b) and *Fusarium* (c) for samples collected form healthy (H) and diseased (D) fields. The significance of the difference was determined by the t-test. ($P < 0.05$, *; $P < 0.01$, **; $P < 0.001$, ***)

Fig.S4 The relationship between *Fusarium* (ZFOTU_585) and other microorganisms in the bacteria-fungi co-occurrence networks.

Table S1 Information of rhizosphere soil collection sample.

Table.S2 Dominant groups of diseased soil and healthy soil samples screened by LEfSe analysis (LDA > 3.5 and remove unclassified microbiome)

Table.S3 PERMANOVA analysis using the bray-curtis distances for FWD and sampling site in beta diversity.

Table.S4 Topology properties of the intra- and inter-kingdom networks.

Table.S5 The taxonomic position of top 10 hubs in intra- and interkingdom networks

1 Supplementary figures

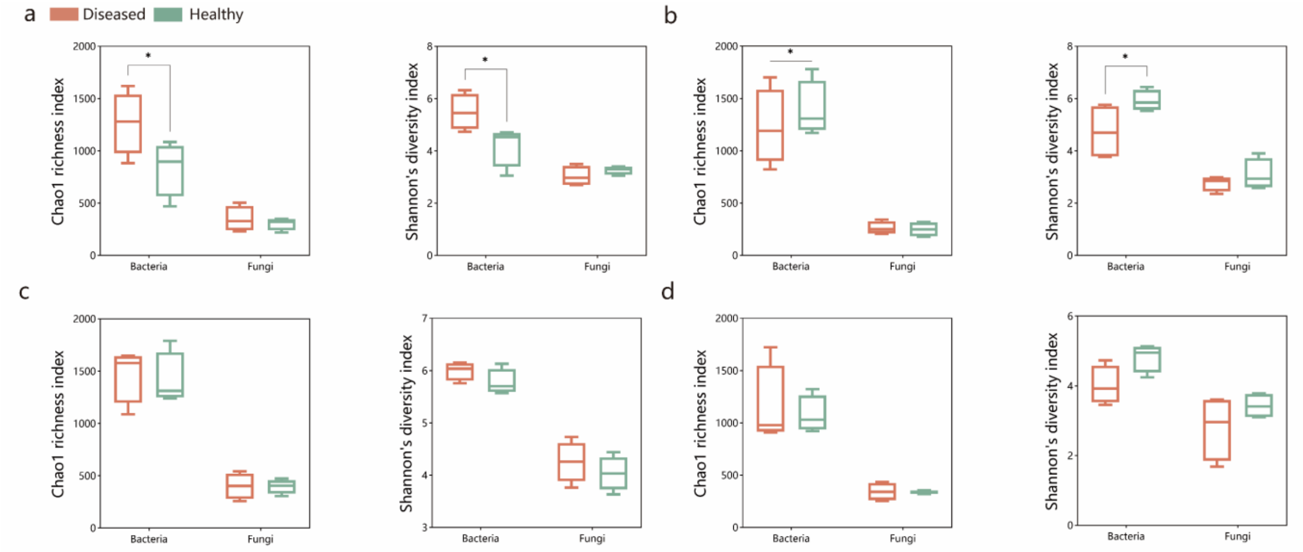

Fig.S1 Chao1 richness index and Shannon's diversity index of bacterial and fungal communities affected by FWD in the different sampling site. a. microbial α-diversity analysis in the Dragon King Ridge, Shangluo. (The Chao1 richness index and Shannon's diversity index of bacterial community in healthy plot was significantly higher than that in diseased plot) b. microbial α-diversity analysis in the Huamiao Village, Shangluo. (The Chao1 richness index and Shannon's diversity index of bacterial community in diseased plot was significantly higher than that in healthy plot)c. microbial α-diversity analysis in the Sanmenxia. d. microbial α-diversity analysis in the Neixiang.

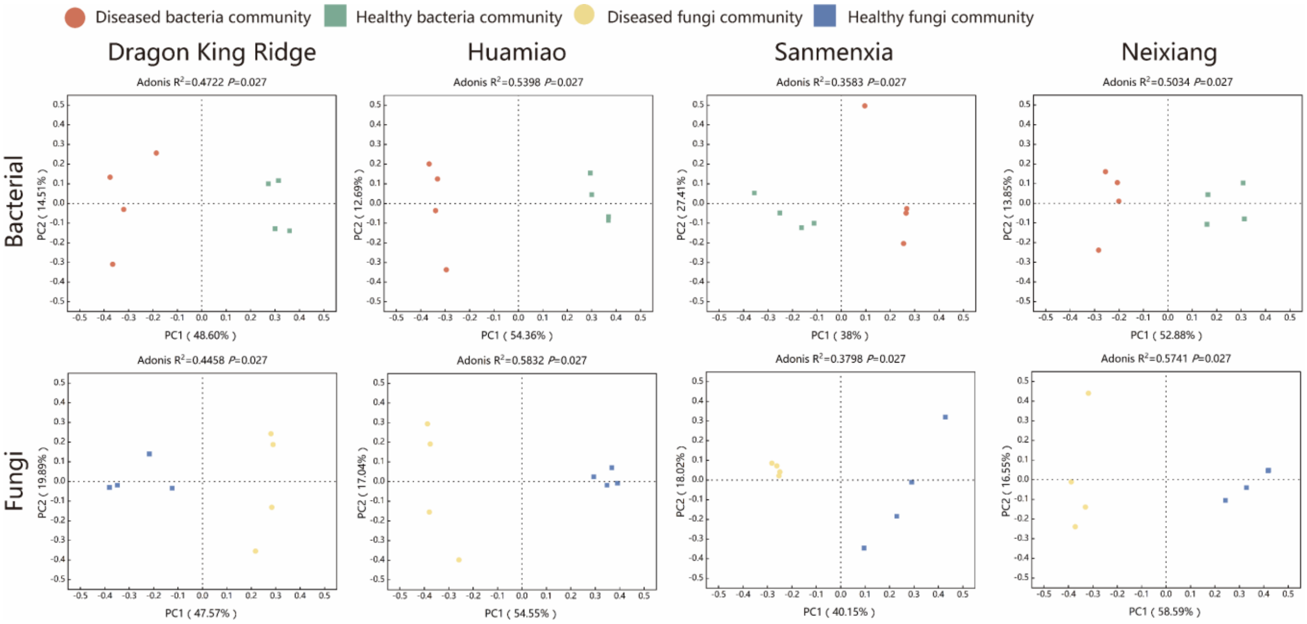

Fig.S2 Principal coordinates analysis (PCoA) ordinations of Bray–Cutis dissimilarity matrices showing a significant association of the bacterial (up) and fungi (under) disease and health community composition in different sites.

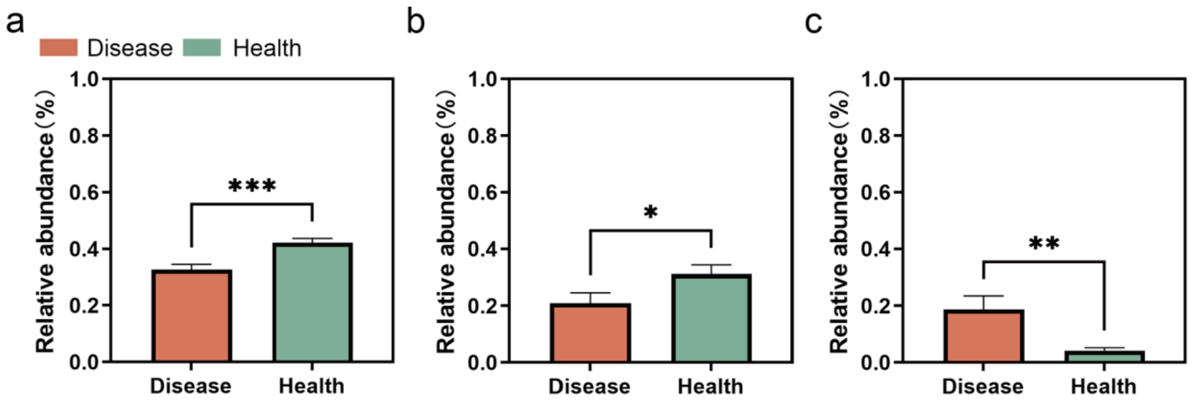

Fig.S3 The percents of Prteobacteria (a), Basidiomycota (b) and *Fusarium* (c) for samples collected form healthy (H) and diseased (D) fields. The significance of the difference was determined by the t-test. ($P < 0.05$, *; $P < 0.01$, **; $P < 0.001$, ***)

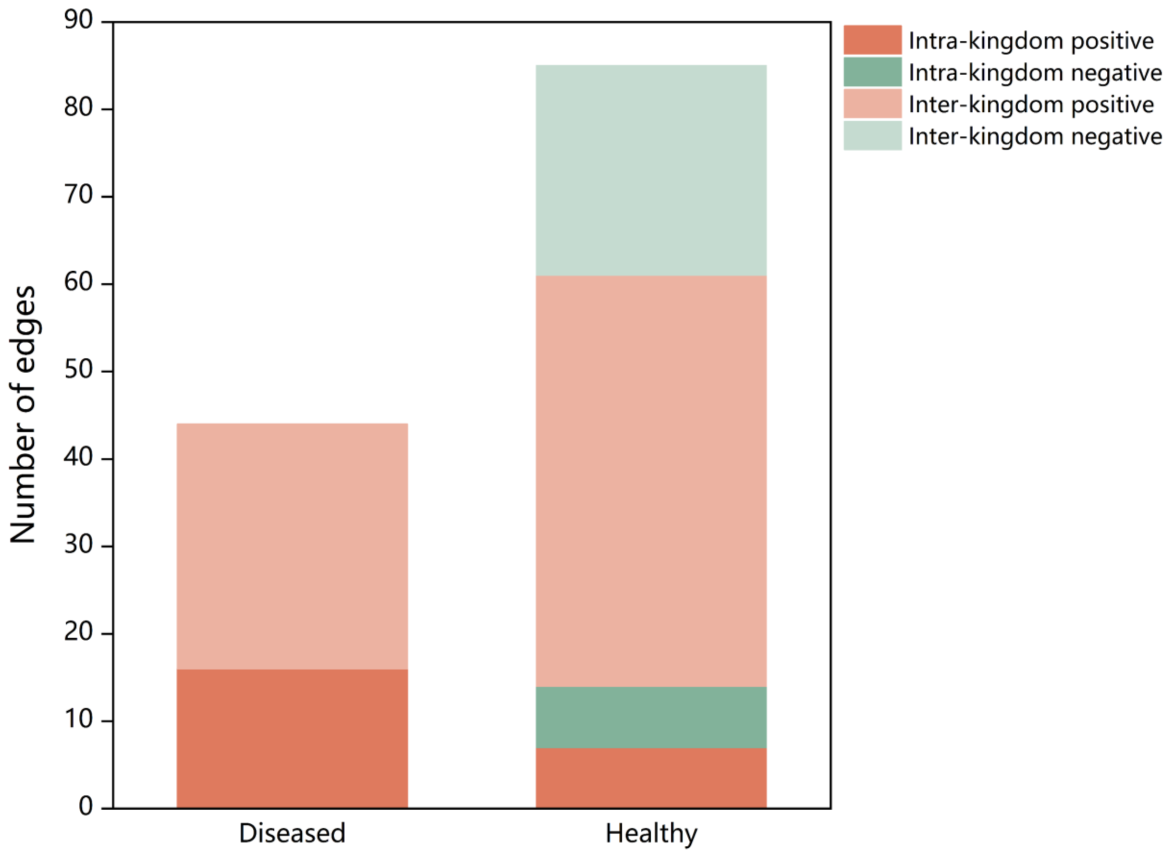

Fig.S4 The relationship between *Fusarium* (ZFOTU_585) and other microorganisms in the bacteria-fungi co-occurrence networks

2 Supplementary tables

Table S1 Origins and distribution of rhizosphere soil samples.

Name	Sample	Location	Latitude	Longitude	Altitude	DI[a]	DS[b]
SLD -LWL	Diseased	Shangluo	110°11’54.66”	34°11’46.10”	1070.4m	29.00 ±4.00	0.14± 0.03
SLH -LWL	Healthy	Shangluo	110°11’58.14”	34°11’44.60”	1078.5m		
SLD -HM	Diseased	Shangluo	110°12’35.11”	34°12’28.68”	1102.5m	40.00 ±8.22	0.20± 0.06
SLH -HM	Healthy	Shangluo	110°12’34.34”	34°12’29.28”	1109.2m		
SMXD	Diseased	Sanmenxia	111°0’16.72”	34°5’47.54”	872.6m	42.00 ±7.52	0.26± 0.04
SMXH	Healthy	Sanmenxia	111°0’13.15”	34°5’29.88”	802.5m		
NXD	Diseased	Nanyang	111°49’25.25”	33°8’50.92”	149.7m	26.00 ±5.79	0.16± 0.05
NXH	Healthy	Nanyang	111°49’3.42”	33°8’59.68”	149.7m		

[a] Disease incidence, [b] Disease severity.

Table.S2 Dominant groups of diseased soil and healthy soil samples screened by LEfSe analysis (LDA ＞ 3.5 and remove unclassified microbiome)

Microbial communities	Species	Group	Mean	LDA value	*P* value
Bacterial community	Flavobacteriales	Disease	4.58	4.04	0.042
	Rhizobiales	Health	5.31	4.39	0.013
	Proteobacteria	Health	5.62	4.63	0.001
	Allorhizobium-Neorhizobium-Pararhizobium-Rhizobium	Health	5.04	4.18	0.046
	Sphingomonadales	Health	4.95	4.14	0.012
	Sphingomonadaceae	Health	4.95	4.14	0.012
	Sphingobium	Health	4.5	3.91	0.005
	Ensifer	Health	4.37	3.84	0.004
	Alphaproteobacteria	Health	5.49	4.57	0.002
Fungal community	Ascomycota	Disease	5.84	4.8	0.012
	Hypocreales	Disease	5.46	4.87	0.038
	Aspergillaceae	Disease	5.1	4.6	0.019
	Fusarium	Disease	5.27	4.86	0.000
	Penicillium	Disease	4.86	4.41	0.006
	Phaeosphaeriaceae	Disease	4.19	3.58	0.014
	Eurotiales	Disease	5.14	4.58	0.042
	Nectriaceae	Disease	5.43	4.94	0.000
	Plectosphaerella	Disease	4.76	4.18	0.018

Sordariomycetes	Disease	5.68	4.91	0.006
Mrakiaceae	Health	5.25	4.42	0.018
Tausonia	Health	5.25	4.42	0.018
Barnettozyma	Health	4.65	4.33	0.036
Basidiomycota	Health	5.49	4.69	0.005
Saccharomycetes	Health	4.67	4.34	0.007
Saccharomycetales	Health	4.67	4.34	0.007
Thielavia	Health	4.05	3.6	0.018
Leotiomycetes	Health	4.3	3.73	0.000
Piskurozymaceae	Health	5.01	4.31	0.004
Filobasidiales	Health	5.03	4.32	0.002
Tremellomycetes	Health	5.45	4.65	0.008
Solicoccozyma	Health	5.01	4.31	0.004
Cystofilobasidiales	Health	5.25	4.42	0.018
Phaffomycetaceae	Health	4.67	4.34	0.008

Table.S3 PERMANOVA analysis using the bray-curtis distances for FWD and sampling site in beta diversity. （PERMANOVA by adonis of all bacterial 16S and fungal ITS samples）

Microbial communities	Variables	Df[a]	SumsOfSqs[b]	MeanSqs[c]	F.Model	R^2	Pr(>F)[d]
Bacterial community	sampling site	2	0.94	0.47	4.40	0.23	0.001
	FWD	1	0.20	020	1.86	0.05	0.071
	Residuals	28	2.98	0.11	-	0.72	-
Fungal community	sampling site	2	1.47	0.73	4.91	0.23	0.001
	FWD	1	0.84	0.84	5.66	0.13	0.001
	Residuals	28	4.18	0.15	-	0.64	-

[a] degrees of freedom, [b] sum of squares, [c] mean sum of squares, [d] p-values are based on 999 permutations with subsequent Bonferroni correction.

Table.S4 Topology properties of the intra- and inter-kingdom networks.

Name	Bacterial diseased	Bacterial healthy	fungal diseased	fungal healthy
nodes	647	702	193	213

edges	6831	12023	1472	2076
positive	6671	10308	1449	2008
negative	160	1715	23	68
Average degree	21.12	34.25	15.25	19.49
Average path length	4.48	3.50	4.33	4.06
Network diameter	13.30	13.95	11.37	11.17
Network density	0.03	0.05	0.079	0.09
Clustering coefficient	0.78	0.60	0.88	0.91

Table.S5 The taxonomic position of top 10 hubs in intra- and interkingdom networks.

ZOTUs in intra-kingdom	Taxonomic position

networks	
ZBOTU_701	Bacteria—Actinobacteriota—Thermoleophilia—Gaiellales--norank_o__Gaiellales--norank_f__norank_o__Gaiellales--unclassified_g__norank_f__norank_o__Gaiellales
ZBOTU_83	Bacteria—Myxococcota--bacteriap25--norank_c__bacteriap25--norank_o__norank_c__bacteriap25--norank_f__norank_o__norank_c__bacteriap25--unclassified_g__norank_f__norank_o__norank_c__bacteriap25
ZBOTU_1567	Bacteria—Proteobacteria—Gammaproteobacteria—Burkholderiales--Nitrosomonadaceae MND1--unclassified_g__MND1
ZBOTU_606	Bacteria—Actinobacteriota—Actinobacteria—Corynebacteriales—Nocardiaceae—Rhodococcus--unclassified_g__Rhodococcus
ZBOTU_20750	Bacteria—Actinobacteriota—Rubrobacteria—Rubrobacterales—Rubrobacteriaceae—Rubrobacter--metagenome_g__Rubrobacter
ZBOTU_315	Bacteria—Actinobacteriota—Thermoleophilia—Gaiellales—Gaiellaceae—Gaiella--unclassified_g__Gaiella

ZBOTU_3418	Bacteria—Actinobacteriota—Acidimicrobiia--unclassified_c__Acidimicrobiia--unclassified_c__Acidimicrobiia--unclassified_c__Acidimicrobiia--unclassified_c__Acidimicrobiia-
ZBOTU_1709	Bacteria—Actinobacteriota—Actinobacteria—Micromonosporales--Micromonosporaceae--unclassified_f__Micromonosporaceae--unclassified_f__Micromonosporaceae
ZBOTU_31803	Bacteria—Actinobacteriota—Thermoleophilia—Gaiellales--norank_o__Gaiellales--norank_f__norank_o__Gaiellales--uncultured_microorganism_g__norank_f__norank_o__Gaiellales
ZBOTU_22522	Bacteria—Bacteroidota—Bacteroidia—Chitinophagales—Chitinophagaceae--unclassified_f__Chitinophagaceae--unclassified_f__Chitinophagaceae
ZBOTU_589	Bacteria—Acidobacteriota—Blastocatellia—Pyrinomonadales--Pyrinomonadaceae RB41--uncultured_Acidobacteriaceae_bacterium_g__RB41
ZBOTU_603	Bacteria—Acidobacteriota—Vicinamibacteria—Vicinamibacterales—Vicinamibacteraceae--norank_f__Vicinamibacteraceae--unclassified_g__norank_f__Vicinamibacteraceae
ZBOTU_664	Bacteria—Bacteroidota—Bacteroidia—Cytophagales—Microscillaceae--norank_f__Microscillaceae--unclassified_g__norank_f__Microscillaceae

ZBOTU_411	Bacteria—Proteobacteria—Gammaproteobacteria—Burkholderiales--SC-I-84--norank_f__SC-I-84--unclassified_g__norank_f__SC-I-84
ZBOTU_466	Bacteria—Proteobacteria—Alphaproteobacteria—Rhizobiales---Rhizobiales_Incertae_Sedis--Nordella unclassified_g__Nordella
ZBOTU_818	Bacteria—Planctomycetota—Phycisphaerae—Tepidisphaerales--WD2101_soil_group--norank_f__WD2101_soil_group--uncultured_planctomycete_g__norank_f__WD2101_soil_group
ZBOTU_607	Bacteria—Proteobacteria—Alphaproteobacteria—Rhizobiales---Beijerinckiaceae—Microvirga--unclassified_g__Microvirga
ZBOTU_488	Bacteria—Acidobacteriota—Blastocatellia—Pyrinomonadales—Pyrinomonadaceae--RB41--unclassified_g__RB41
ZBOTU_417	Bacteria—Acidobacteriota—Blastocatellia—Blastocatellales—Blastocatellaceae--unclassified_f__Blastocatellaceae---unclassified_f__Blastocatellaceae
ZBOTU_389	Bacteria—Actinobacteriota—Actinobacteria—Micrococcales—Microbacteriaceae—Agromyces--unclassified_g__Agromyces

ZFOTU_1595	Fungi—Ascomycota—Eurotiomycetes—Eurotiales—Aspergillaceae—Penicillium--unclassified_g__Penicillium
ZFOTU_2277	Fungi—Ascomycota—Sordariomycetes—Sordariales—Chaetomiaceae—Chaetomium--Chaetomium_sp
ZFOTU_2252	Fungi—Ascomycota—Sordariomycetes—Hypocreales—Hypocreaceae—Trichoderma--Trichoderma_polysporum
ZFOTU_2247	Fungi—Ascomycota—Eurotiomycetes—Eurotiales—Aspergillaceae—Penicillium--Penicillium_rubidurum
ZFOTU_2267	Fungi—Ascomycota—Eurotiomycetes—Eurotiales—Aspergillaceae—Penicillium--unclassified_g__Penicillium
ZFOTU_1577	Fungi—Ascomycota—Eurotiomycetes—Eurotiales—Aspergillaceae—Penicillium--Penicillium_ochrochloron
ZFOTU_1793	Fungi—Ascomycota--Dothideomycetes --Botryosphaeriales—Botryosphaeriaceae—Macrophomina--Macrophomina_phaseolina

ZFOTU_2257	Fungi—Ascomycota—Sordariomycetes—Sordariales—Lasiosphaeriaceae—Echria--Echria_sp
ZFOTU_10289	Fungi—Ascomycota—Eurotiomycetes—Chaetothyriales--unclassified_o__Chaetothyriales--unclassified_o__Chaetothyriales--unclassified_o__Chaetothyriales
ZFOTU_2240	Fungi--Ascomycota—Sordariomycetes—Sordariales—Chaetomiaceae---unclassified_f__Chaetomiaceae--unclassified_f__Chaetomiaceae
ZFOTU_4508	Fungi—Ascomycota—Sordariomycetes—Hypocreales--unclassified_o__Hypocreales--unclassified_o__Hypocreales--unclassified_o__Hypocreales
ZFOTU_2290	Fungi—Ascomycota—Sordariomycetes—Hypocreales--Hypocreales_fam_Incertae_sedis—Acremonium--Acremonium_acutatum
ZFOTU_4491	Fungi—Ascomycota—Sordariomycetes—Hypocreales---Nectriaceae--unclassified_f__Nectriaceae--Nectriaceae_sp
ZFOTU_22635	Fungi—Mortierellomycota--Mortierellomycetes—Mortierellales—Mortierellaceae—Mortierella--Mortierella_simplex

ZFOTU_4431	Fungi-- unclassified_k__Fungi
ZFOTU_10425	Fungi-- unclassified_k__Fungi
ZFOTU_4438	Fungi-- unclassified_k__Fungi
ZFOTU_4681	Fungi-- unclassified_k__Fungi
ZFOTU_13475	Fungi-- unclassified_k__Fungi
ZFOTU_1553	Fungi-- unclassified_k__Fungi
ZOTUs in interki	Taxonomic position

ngdom networks	
ZBOTU_111	Bacteria-- Crenarchaeota --Nitrososphaeria—Nitrososphaerales—Nitrososphaeraceae--norank_f__Nitrososphaeraceae--uncultured_euryarchaeote_g__norank_f__Nitrososphaeraceae
ZBOTU_22522	Bacteria—Bacteroidota—Bacteroidia—Chitinophagales—Chitinophagaceae--unclassified_f__Chitinophagaceae--unclassified_f__Chitinophagaceae
ZBOTU_606	Bacteria—Actinobacteriota—Actinobacteria—Corynebacteriales---Nocardiaceae—Rhodococcus--unclassified_g__Rhodococcus
ZBOTU_1194	Bacteria—Acidobacteriota—Vicinamibacteria—Vicinamibacterales--norank_o__Vicinamibacterales--norank_f__norank_o__Vicinamibacterales--unclassified_g__norank_f__norank_o__Vicinamibacterales
ZBOTU_473	Bacteria—Bacteroidota—Bacteroidia—Chitinophagales—Chitinophagaceae--Puia unclassified_g__Puia

ZBOTU_20750	Bacteria—Actinobacteriota—Rubrobacteria—Rubrobacterales—Rubrobacteriaceae—Rubrobacter--metagenome_g__Rubrobacter
ZBOTU_1256	Bacteria-- Proteobacteria --Gammaproteobacteria—Burkholderiales—Oxalobacteraceae--unclassified_f__Oxalobacteraceae--unclassified_f__Oxalobacteraceae
ZBOTU_11341	Bacteria—Acidobacteriota—Vicinamibacteria—Vicinamibacterales--norank_o__Vicinamibacterales--norank_f__norank_o__Vicinamibacterales--unclassified_g__norank_f__norank_o__Vicinamibacterales
ZBOTU_411	Bacteria-- Proteobacteria --Gammaproteobacteria—Burkholderiales--SC-I-84--norank_f__SC-I-84--unclassified_g__norank_f__SC-I-84
ZBOTU_589	Bacteria—Acidobacteriota—Blastocatellia—Pyrinomonadales--Pyrinomonadaceae RB41--uncultured_Acidobacteriaceae_bacterium_g__RB41
ZBOTU_628	Bacteria—Acidobacteriota—Blastocatellia—Blastocatellales—Blastocatellaceae--unclassified_f__Blastocatellaceae--unclassified_f__Blastocatellaceae
ZBOTU_607	Bacteria-- Proteobacteria --Alphaproteobacteria—Rhizobiales—Beijerinckiaceae—Microvirga--unclassified_g__Microvirga

ZBOTU_603	Bacteria--Proteobacteria--Deltaproteobacteria--Bdellovibrionales--Bdellovibrionaceae--OM27 clade
ZBOTU_288	Bacteria--Proteobacteria--Deltaproteobacteria--Bdellovibrionales--Bdellovibrionaceae--OM27 clade
ZBOTU_607	Bacteria--Proteobacteria--Deltaproteobacteria--Bdellovibrionales--Bdellovibrionaceae--OM27 clade
ZBOTU_603	Bacteria—Acidobacteriota—Vicinamibacteria—Vicinamibacterales—Vicinamibacteraceae--norank_f__Vicinamibacteraceae--unclassified_g__norank_f__Vicinamibacteraceae
ZBOTU_288	Bacteria—Actinobacteriota—Actinobacteria—Frankiales—Geodermatophilaceae--unclassified_f__Geodermatophilaceae--unclassified_f__Geodermatophilaceae
ZBOTU_664	Bacteria—Bacteroidota—Bacteroidia—Cytophagales—Microscillaceae--norank_f__Microscillaceae--unclassified_g__norank_f__Microscillaceae
ZBOTU_389	Bacteria—Actinobacteriota—Actinobacteria—Micrococcales—Microbacteriaceae—Agromyces--unclassified_g__Agromyces

ZBOTU_344	Bacteria—Acidobacteriota—Blastocatellia—Blastocatellales--Blastocatellaceae—Stenotrophobacter--uncultured_soil_bacterium_g__Stenotrophobacter
ZBOTU_222	Bacteria-- Proteobacteria --Gammaproteobacteria—Xanthomonadales—Xanthomonadaceae--unclassified_f__Xanthomonadaceae--unclassified_f__Xanthomonadaceae
ZFOTU_585	Fungi--Ascomycota --Sordariomycetes--Hypocreales--Nectriaceae--Fusarium--*Fusarium*.nematophilum
ZFOTU_4304	Fungi—Mortierellomycota--Mortierellomycetes—Mortierellales—Mortierellaceae—Mortierella--Mortierella_alpina
